# Is induction of fetal asystole associated with adverse health outcomes during second‐trimester termination induction of labor?

**DOI:** 10.1002/pmf2.70177

**Published:** 2025-11-13

**Authors:** Neha G. Reddy, Vanya Manthena, Jocelyn Wascher, Eryn Wanyonyi, Camille A. Johnson, Lahari Vuppaladhadiam, Julie Chor, Beth A. Plunkett, Isa Ryan, Olivert Mbah, Jungeun Lee, Emily Barker, Laura Laursen, Leanne R. McCloskey, Sloane L. York, Ashish Premkumar

**Affiliations:** ^1^ Department of Obstetrics & Gynecology Biological Sciences Division, The University of Chicago Chicago Illinois USA; ^2^ Department of Obstetrics & Gynecology Endeavor Health Evanston Illinois USA; ^3^ Department of Obstetrics & Gynecology Washington University School of Medicine in St. Louis St. Louis Missouri USA; ^4^ Department of Obstetrics & Gynecology Rush University School of Medicine Chicago Illinois USA; ^5^ Department of Obstetrics & Gynecology Feinberg School of Medicine, Northwestern University Chicago Illinois USA

**Keywords:** abortion, induced fetal death, induced labor, pregnancy second trimester

## Abstract

**Objective:**

To evaluate whether induction of fetal asystole (IFA) during second‐trimester termination induction of labor (2T termination IOL) is associated with duration of labor and adverse health outcomes.

**Study design:**

We analyzed a retrospective cohort of individuals undergoing second‐trimester termination IOL (2T termination IOL) between 14 0/7 to 27 6/7 weeks of gestation of a singleton at four centers from 2009 to 2019. Individuals with prelabor rupture of membranes, preterm labor, intrauterine fetal demise, or cervical insufficiency were excluded. The primary exposure was IFA. The primary outcome was duration of labor, treated continuously and dichotomously (i.e., upper quartile of the duration of labor for the cohort vs. all other quartiles). The secondary outcomes were composite morbidity and its components (uterine rupture, need for blood transfusion, clinical chorioamnionitis, intensive care unit admission, or need for readmission). Bivariate and multivariate analyses were performed, comparing individuals who underwent IFA to individuals who had fetal cardiac activity (FCA) at the time of 2T termination IOL. A priori, the receipt of mifepristone was chosen as a confounding covariate. A Kaplan–Meier curve was generated, censoring at the time of delivery. Subgroup analyses for the primary outcome were performed based on parity. A sensitivity analysis was conducted, with the use of propensity score matching, to assess for differences in all outcomes.

**Results:**

A total of 358 participants were analyzed of which 83 (20.8%) underwent IFA with potassium chloride and 275 (76.8%) had FCA. There were no significant differences in the duration of labor between the two groups (13.1 h in the IFA group vs. 12.0 h in the FCA group, *p* = 0.84) or the frequency of experiencing the highest quartile of labor duration (26.5% vs. 24.7%, *p* = 0.70).

There was also no difference in the composite secondary outcome (21.7% vs. 17.4%, *p* = 0.42) or its components (i.e., uterine rupture (*p* = 0.053), need for blood transfusion (*p* = 0.10), clinical chorioamnionitis (*p* = 0.96), ICU admission (*p* = 0.41), or need for hospital readmission (*p* = 1.0)). A similar pattern was noted in the sensitivity analysis. On subgroup analysis, there was no difference in the duration of labor based on parity.

**Conclusion:**

IFA is not associated with increased duration of labor when compared to FCA during 2T termination IOL. It is also not associated with adverse outcomes including uterine rupture, need for blood transfusion, clinical chorioamnionitis, ICU admission, or need for hospital admission.

## 1 INTRODUCTION

Induction of fetal asystole (IFA) may be used to ensure fetal demise before termination induction of labor (IOL) in the second trimester (2T termination IOL) to prevent the rare occurrence of delivering a fetus with cardiorespiratory activity [1]. IFA is most often used in situations of patient preference or based on institutional policies necessitating its use above a certain gestational duration. Research has demonstrated that without IFA, there is up to an 11% frequency of cardiac activity at the time of fetal expulsion [2].

Existing data are unclear regarding the impact of IFA on the duration of labor and other health outcomes during 2T termination IOL. Longer duration of labor has separately been demonstrated to be associated with poor maternal health outcomes [3]. Some data demonstrate that the use of IFA is associated with an increased risk of adverse health outcomes for the pregnant individual, including longer durations of labor [4]. Other data found that IFA may actually decrease the duration of labor and cumulative doses of misoprostol given during medication abortion [5].

The decision to pursue IFA at particular gestational ages is institution‐specific and based on shared decision‐making between providers and patients. The 2024 joint statement on IFA by the Society of Family Planning (SFP) and the Society of Maternal‐Fetal Medicine (SMFM) states that further research is needed to assess the clinical outcomes of IFA during 2T termination IOL [1]. The primary objective of our study was to evaluate the relationship between IFA and the duration of labor. The secondary objective was to explore differences in adverse outcomes between individuals who did and did not undergo IFA. We hypothesize that, when compared to individuals with fetal cardiac activity (FCA), IFA during 2T termination IOL does not increase the duration of labor or frequency of adverse health outcomes.

## 2 METHODS

We conducted a retrospective cohort study of a convenience sample of individuals undergoing 2T termination IOL at four tertiary care centers in Cook County, Illinois, from 2009 to 2019. Institutional Review Board approval was obtained from all participating sites. All data were extracted, validated, and deidentified by trained research staff from the electronic medical record and entered into REDCap. Further information about data extraction has previously been reported [6, 7].

People with a singleton pregnancy undergoing 2T termination IOL after IFA or with FCA at the start of IOL were eligible for inclusion. We limited our analysis to individuals between 14 0/7 to 27 6/7 weeks of gestation without evidence of preterm prelabor rupture of membranes (PPROMs), preterm labor (PTL), advanced cervical dilation (ACD), intrauterine fetal demise, or missing outcomes data. Given the relationship between PPROM, PTL, and ACD with clinical chorioamnionitis [8, 9], as well as the relationship between higher‐order gestations and adverse peripartum outcomes [10], including individuals with these diagnoses in our cohort would unduly bias our sample toward a higher probability of our primary composite outcome.

IFA was performed by a trained subspecialist (i.e., maternal‐fetal medicine or complex family planning attendings and fellows) prior to IOL, specifically using potassium chloride (KCl). Ten milli‐equivalents per milliliter of KCl was introduced either into the fetal heart or thorax with an 18‐ or 20‐gauge spinal needle under continuous ultrasound guidance, with a maximum of 30 mL of KCl used in total. Timing of IFA in relation to the IOL was left to the discretion of the treating clinician. To begin the IOL process, prior to misoprostol, use of mifepristone (200 mg/os) or use of mechanical dilation (e.g., laminaria, intracervical balloon catheter) was determined by the treating clinician. All sites adhered to vaginal misoprostol regimens, with dosing changes based on prior uterine scarring, endorsed by SFP and ACOG [11, 12].

The primary exposure was IOL with IFA compared with FCA at the time of misoprostol administration. The primary outcome was duration of labor, defined as the time from the start of the IOL via misoprostol or mechanical dilation (laminaria, intracervical balloon catheter) placement to the expulsion of the fetus in hours, analyzed both categorically (i.e., labor duration in the upper quartile, or highest 25%, of labor duration for the cohort) and continuously. As discussed in other analyses of this dataset, we did not evaluate the time to placental expulsion because of the heterogeneity in diagnosing and managing a retained placenta [3].

The secondary outcomes were composite morbidity (i.e., uterine rupture, need for blood transfusion, clinical chorioamnionitis—as defined by the American College of Obstetricians and Gynecologists [13])—intensive care unit admission, or need for hospital readmission) and its components. The secondary composite outcome was defined as all components of the composite morbidity outcome except clinical chorioamnionitis, given the overlap between maternal fever associated with clinical chorioamnionitis and the side‐effect profile of high‐dose misoprostol [14, 15]. Further secondary outcomes included each individual component of the composite morbidity outcome. All outcomes were in line with the Standardizing Abortion Research Outcomes guidelines [16].

Chi‐squared or Fisher's exact test was performed for categorical variables, and Wilcoxon rank‐sum test was performed for continuous variables. Unadjusted and adjusted quantile regression analyses were performed for the primary outcome, after a Kolmogorov–Smirnov test demonstrated a non‐parametric distribution of the data. An a priori covariate selected for inclusion in the model was receiving mifepristone, given its relationship with reducing the duration of labor [17]. For the secondary outcomes, adjusted and unadjusted relative risks were computed using a regression model with a Poisson distribution in Stata (StataCorp). The results of the regression models are presented as Model 1. A sensitivity analysis was performed using propensity scoring, with nearest neighbor matching based on history of uterine scarring, receipt of mifepristone, self‐reported maternal race/ethnicity, gestational duration, and site of delivery. Results of the sensitivity analysis are presented as Model 2.

For the primary outcome, treated continuously, Kaplan–Meier curves were generated, censoring at the time of fetal expulsion. Curves were compared using the log‐rank test. A Cox proportional hazards assumption was tested, and a model was subsequently generated after no violations to the proportionality assumption were demonstrated. We performed planned subgroup analyses based on parity, given previous data showing an independent association between parity and duration of labor [3].

A *p*‐value of less than 0.05 was set to indicate statistical significance. All analyses were performed using Stata and adhered to the Strengthening the Reporting of Observational Studies in Epidemiology reporting guidelines [18].

## 3 RESULTS

**FIGURE 1:**
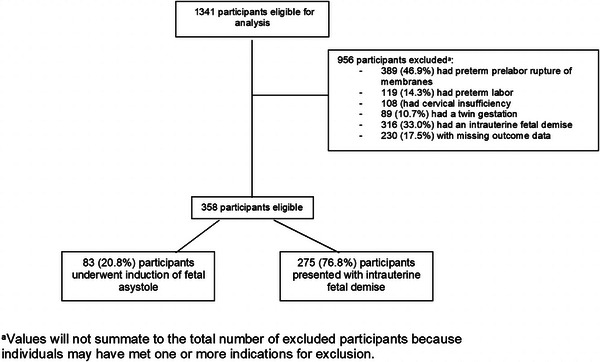
Participant inclusion and exclusion.

Of the original cohort of 1341 individuals available for analysis, 956 individuals were excluded due to PPROM, PTL, cervical insufficiency, twin gestation, intrauterine fetal demise, or missing outcome data (Figure 1). Therefore, 358 participants were analyzed; 83 (20.8%) underwent IFA with KCl and 275 (76.8%) had FCA (Table 1).

**TABLE 1:** Biomedical and sociodemographic data of pregnant people undergoing second‐trimester induction of labor (*n* = 358).

	IFA (*n* = 83)	Fetal cardiac activity present (*n* = 275)	*p* value^a^
Age (years)	32 (28–35)	32 (27–36)	0.74
Race/ethnicity			
NHW	36 (43.4)	102 (37.1)	**0.001**
NHB	6 (7.2)	71 (25.8)	
Latinx	13 (15.7)	47 (17.1)	
AAPI	5 (6.0)	16 (5.8)	
Other or not defined	23 (27.7)	39 (14.2)	
Gestational age (weeks)	23 (22–23)	22 (20–23)	**<0.001**
Body mass index (kg/m^2^)^b^	27.1 (23.8–32.1)	27.1 (23.8–32.3)	0.88
Nulliparous	28 (33.7)	96 (34.9)	0.84
History of uterine scarring^c^			
None	60 (72.3)	233 (84.7)	**0.02**
1 scar	19 (22.9)	31 (11.3)	
2 or more scars	4 (4.8)	11 (4.0)	
Location of delivery			
Site #1	6 (7.2)	55 (20.0)	**<0.001**
Site #2	68 (82.0)	75 (27.3)	
Site #3	1 (1.2)	73 (26.6)	
Site #4	8 (9.6)	72 (26.2)	
Mifepristone administered	66 (78.5)	115 (41.8)	**<0.001**
Method of induction of labor			
Misoprostol	74 (89.2)	235 (85.5)	0.51
Intracervical balloon catheter	0 (0.0)	5 (1.8)	
Oxytocin	1 (1.2)	11 (4.0)	
Artificial rupture of membranes	7 (8.4)	17 (6.2)	
Laminaria	1 (1.2)	7 (2.5)	
Total misoprostol administered (µg)^d^	1800 (1600–2400)	1600 (1200–2400)	0.07

*Note*: Data are median (interquartile range) or *n* (%) unless otherwise specified. Bold indicates statistical significance <0.05.

Abbreviations: AAPI, Asian American/Pacific Islander; IFA, induction of fetal asystole; NHB, Non‐Hispanic Black; NHW, Non‐Hispanic White.

^a^ Chi‐squared or Fisher's exact test for categorical variables, Wilcoxon rank‐sum test for continuous variables.

^b^ Available for 291 participants.

^c^ Prior uterine scar includes prior low transverse; classical, t‐shaped, or unknown hysterotomy.

^d^ Available for 358 participants.

On bivariate analysis, the distribution of maternal race/ethnicity was significantly different between groups. Individuals undergoing IFA were less likely to self‐identify as non‐Hispanic Black (7.2%) compared with FCA group (25.8%, *p* = 0.001). Individuals undergoing IFA presented approximately 1 week further in gestation than those with FCA (23 vs. 22 weeks; *p* < 0.001). There were no statistical differences in age of participants (32 in both groups; *p* = 0.74), body mass index (27.1 in both groups; *p* = 0.88), or frequency of nulliparity (33.7% in IFA group vs. 34.9% in FCA group, *p* = 0.84).

The distribution of the number of prior uterine scars differed between groups (*p* = 0.02); the proportion of individuals with no uterine scarring was higher in the FCA group (84.7%) compared to the IFA group (72.3%). Individuals who underwent IFA had a higher frequency of receiving mifepristone (78.5% in IFA group vs. 41.8% in FCA group; *p* < 0.001), though total misoprostol given was not significantly different (1800 µg in IFA group vs. 1600 µg in FCA group; *p* = 0.51, Table 1).

On unadjusted analyses, there was no significant difference in duration of labor between the two groups (13.1 h in IFA group vs. 12.0 h in FCA group; *p* = 0.84) nor in the probability of experiencing the upper quartile of labor duration, which was 18.5 h (26.5% in IFA group vs. 24.7% in FCA group; *p* = 0.74). There was also no statistical difference in overall composite morbidity between the groups (10.8% in IFA group vs. 5.4% in FCA group; *p* = 0.13). These findings remained true when looking at the individual components of the composite morbidity, including rates of uterine rupture (2.4% in IFA group vs. 0.0% in FCA group; *p* = 0.053), blood transfusion (7.2% in IFA group vs. 2.9% in FCA group; *p* = 0.10), clinical chorioamnionitis (13.2% in IFA group vs. 13.4% in FCA group; *p* = 0.96), intensive care unit admission (1.2% in IFA group vs. 0.4% in FCA group; *p* = 0.41), and need for hospital readmission (2.4% in IFA group vs. 2.2% in FCA group; *p* = 1.0, Table 2).

**TABLE 2:** The relationship between etiology of fetal death and duration of labor and composite morbidity among people undergoing second‐trimester induction of labor.

	IFA (*n* = 83)	Fetal cardiac activity present (*n* = 275)	*p* value^a^
Duration of labor (hours)	13.1 (8.8–18.9)	12.0 (8.6–18.2)	0.84
Experiencing the upper quartile of labor duration (i.e., 18.5 h)^b^	22 (26.5)	68 (24.7)	0.74
Primary composite outcome^c^	18 (21.7)	48 (17.4)	0.38
Composite outcome without clinical chorioamnionitis	9 (10.8)	15 (5.4)	0.13
Components of primary composite outcome^c^			
Uterine rupture^d^	2 (2.4)	0 (0.0)	0.053
Need for blood transfusion^e^	6 (7.2)	8 (2.9)	0.10
Clinical chorioamnionitis	11 (13.2)	37 (13.4)	0.96
Intensive care unit admission	1 (1.2)	1 (0.4)	0.41
Need for hospital readmission	2 (2.4)	6 (2.2)	1.0

*Note*: Data are median (interquartile range) or *n* (%) unless otherwise specified. Bold indicates statistical significance <0.05.

Abbreviation: IFA, induction of fetal asystole.

^a^ Chi‐squared or Fisher's exact test for categorical variables.

^b^ Quartiles of duration of labor were derived from the index cohort, rather than another dataset.

^c^ Defined as one or more of the following: need for a blood transfusion, clinical chorioamnionitis, intensive care unit admission, uterine rupture, and need for readmission. Participants could meet one or more of the outcomes associated with the primary composite outcome. Therefore, frequencies may not summate to the total sample size meeting the primary composite outcome.

^d^ Both uterine ruptures occurred among individuals with two prior uterine scars.

^e^ 14/14 blood transfusions occurred with an estimated blood loss greater than 500 mL.

On regression analyses, there was no significant association between groups and the duration of labor, as well as all other secondary outcomes, in both unadjusted and adjusted models (Table 5, Model 1). On sensitivity analysis using propensity score matching, 63 participants were analyzed in each group. In these matched cohorts, there was no longer a statistical difference in race/ethnicity between the two groups (*p* = 0.21), gestational duration (23 weeks in both groups; *p* = 0.67), frequency of mifepristone administration (46% in IFA group vs. 50% in FCA group; *p* = 0.40), or history of uterine scarring (*p* = 0.18, Table 3). There remained no difference in any of the primary or secondary outcomes (Table 4). Again, on regression analyses, there was no significant association between groups and any of the primary or secondary outcomes (Table 5, Model 2).

**TABLE 3:** Biomedical and sociodemographic data of pregnant people undergoing second‐trimester induction of labor after propensity score matching (*n* = 126).

	IFA (*n* = 63)	Fetal cardiac activity present (*n* = 63)	*p* value^a^
Age (years)	32 (28–36)	32 (28–36)	0.70
Race/ethnicity			
NHW	27 (42.8)	20 (31.8)	0.21
NHB	6 (9.5)	14 (22.2)	
Latinx	11 (17.5)	10 (15.9)	
AAPI	2 (3.2)	5 (7.9)	
Other or not defined	17 (27.0)	14 (22.2)	
Gestational age (weeks)	23 (22–23)	23 (22–23)	0.67
Body mass index (kg/m^2^)^b^	28.1 (24.0–32.8)	26.3 (23.1–31.5)	0.11
Nulliparous	24 (38.1)	25 (39.7)	0.85
History of uterine scarring^c^			
None	45 (71.4)	49 (77.8)	0.18
1 scar	15 (23.8)	8 (12.7)	
2 or more scars	3 (4.8)	6 (9.5)	
Location of delivery			
Site #1	6 (9.5)	20 (31.7)	**<0.001**
Site #2	48 (76.2)	22 (34.9)	
Site #3	1 (1.6)	16 (25.4)	
Site #4	8 (12.7)	5 (7.9)	
Mifepristone administered	46 (73.0)	50 (79.4)	0.40
Method of induction of labor			
Misoprostol	55 (87.3)	53 (84.0)	0.43
Intracervical balloon catheter	0 (0)	2 (3.2)	
Oxytocin	1 (1.6)	2 (3.2)	
Artificial rupture of membranes	6 (9.5)	3 (4.8)	
Laminaria	1 (1.6)	3 (4.8)	
Total misoprostol administered(µg)^d^	2000 (1600–2400)	2000 (1200–2800)	0.63

*Note*: Data are median (interquartile range) or *n* (%) unless otherwise specified. Bold indicates statistical significance <0.05.

Abbreviations: AAPI, Asian American/Pacific Islander; m, meter; µg, micrograms; kg, kilogram; IFA, induction of fetal asystole; NHB, Non‐Hispanic Black; NHW, Non‐Hispanic White.

^a^ Chi‐squared or Fisher's exact test for categorical variables, Kruskal–Wallis test for continuous variables.

^b^ Available for 123 participants.

^c^ Prior uterine scar includes prior low transverse, classical, t‐shaped, or unknown hysterotomy.

^d^ Available for 126 participants.

**TABLE 4:** The relationship between etiology of fetal death and the duration of labor and composite morbidity among people undergoing second‐trimester induction of labor, after propensity score matching.

	IFA (*n* = 83)	Fetal cardiac activity present (*n* = 63)	*p* value^a^
Duration of labor (hours)	13.5 (9.5–19.9)	13.8 (8.3–20.2)	0.93
Experiencing the upper quartile of labor duration (i.e., 18.5 h)^b^	18 (28.6)	20 (31.7)	0.70
Primary composite outcome^c^	15 (23.8)	19 (30.2)	0.42
Composite outcome without clinical chorioamnionitis	8 (12.7)	4 (6.3)	0.36
Components of primary composite outcome^c^			
Uterine rupture^d^	2 (3.2)	0 (0.0)	0.49
Need for blood transfusion^e^	5 (7.9)	2 (3.2)	0.44
Clinical chorioamnionitis	9 (14.3)	17 (27.0)	0.08
Intensive care unit admission	1 (1.6)	0 (0)	1.0
Need for hospital readmission	2 (3.2)	2 (3.2)	1.0

*Note*: Data are median (interquartile range) or *n* (%) unless otherwise specified. Bold indicates statistical significance <0.05.

Abbreviation: IFA, induction of fetal asystole.

^a^ Chi‐squared or Fisher's exact test for categorical variables.

^b^ Quartiles of duration of labor were derived from the index cohort, rather than another dataset.

^c^ Defined as one or more of the following: need for a blood transfusion, clinical chorioamnionitis, intensive care unit admission, uterine rupture, and need for readmission. Participants could meet one or more of the outcomes associated with the primary composite outcome. Therefore, frequencies may not summate to the total sample size meeting the primary composite outcome.

^d^ Both uterine ruptures occurred among individuals with two prior uterine scars.

^e^ 7/7 blood transfusions occurred with an estimated blood loss greater than 500 mL.

**TABLE 5:** Uni‐ and multivariate models.

	MODEL 1				MODEL 2	
	RR (95% CI)	aRR^b^ (95% CI)^a^	ß (95% CI)	aß (95% CI)^a^	RR (95% CI)	ß (95% CI)
Duration of labor (hours)	–	–	−0.51 (−1.70 to 0.66)	−0.46 (−1.62 to 0.71)	–	0.14 (−1.75 to 2.02)
Experiencing the upper quartile of duration of labor	0.97 (0.76–1.22)	0.92 (0.71–1.19)	–	–	1.05 (0.77−1.45)	–
Primary composite outcome	0.89 (0.68–1.18)	0.93 (0.70–1.23)	–	–	1.12 (0.80–1.58)	–
Composite outcome without clinical chorioamnionitis	0.71 (0.47–1.07)	–^b^	–	–	0.71 (0.39–1.29)	–

*Note*: Model 1 uses the entire cohort. Model 2 uses the propensity‐score‐matched cohort. Bold indicates *p* < 0.05.

Abbreviations: aRR, Adjusted Relative Risk; CI, confidence interval; IFA, induction of fetal asystole; RR, Relative Risk.

^a^ Controlling for receipt of mifepristone.

^b^ Due to a low frequency of occurrence, the decision was made to forego multivariate modeling to avoid overfitting.

**FIGURE 2:**
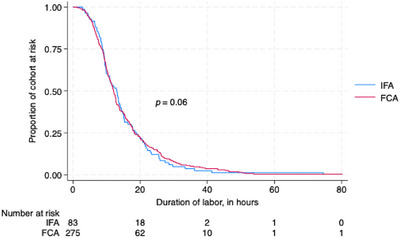
Kaplan–Meier survival estimate of the duration of labor, in hours. FCA, fetal cardiac activity present; IFA, induction of fetal asystole.

**FIGURE 3:**
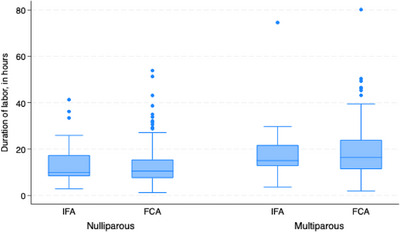
The duration of labor of the cohort, stratified by parity. FCA, fetal cardiac activity present; IFA, induction of fetal asystole. For nulliparous participants, *p* = 0.66, for multiparous patients, *p* = 0.75 on Wilcoxon rank‐sum testing.

On survival analysis, there were no significant differences in the Kaplan–Meier curves (*p* = 0.06, Figure 2). The unadjusted Hazard Ratio (HR) was 0.97 (95% confidence interval [CI], 0.76–1.23), while the adjusted HR was 1.05 (95% CI, 0.81–1.37). On subgroup analysis, the duration of labor was not significantly different between groups when considering both nulliparous and multiparous individuals (Figure 3).

## 4 DISCUSSION

This research builds on the recommendations outlined in the 2024 joint statement released by SFP and SMFM on IFA. The statement [1] outlines a need for further research on clinical outcomes of IFA before uterine evacuation, including “length of procedure, hemorrhage risk, and blood loss.” Based on our analysis, IFA is not associated with an increase in duration of labor when compared to IOL of individuals with FCA. This pattern was seen after controlling for receipt of mifepristone and in both nulliparous and multiparous individuals. IFA also does not appear to be associated with adverse health outcomes,

Longer duration of labor has been associated with adverse health outcomes [3]. Furthermore, longer durations of labor may impact the workflow of labor and delivery units and staffing requirements, thereby potentially influencing access to obstetrical care services. Importantly, in our analysis, IFA was not associated with a difference in duration of labor after adjusting for confounding covariates when compared to people undergoing IOL with FCA. This suggests that IFA is both a cost‐ and time‐effective option for patients who can be employed on labor and delivery without increasing workflow challenges. These findings can help to assist in both setting patient expectations for their experiences on labor and delivery, as well as with nursing and physician staffing and room allocation.

The use of IFA is primarily aimed at cessation of cardiorespiratory activity in the fetus at the time of delivery in the setting of medication abortion [1]. Data are inconsistent related to risks of IFA during medication abortion. A systematic review by Tufa et al. found that use of potassium chloride for IFA may shorten duration of labor while use of digoxin for IFA may be associated with increased complications, such as hospital readmission and signs of infection [4]. Multiple other studies compared people who underwent IFA to those who did not before 2T termination IOL abortion and found no differences in duration of labor or adverse health outcomes [19, 20]. Conversely, Elimian et al. found that IFA significantly reduced duration of labor and cumulative doses of misoprostol compared to those who did not undergo IFA [5].

In this study, IFA was not associated with adverse outcomes when compared to FCA. The specific adverse outcomes analyzed were need for blood transfusion, uterine rupture, clinical chorioamnionitis, intensive care unit admission, or need for hospital readmission. In terms of blood transfusion, the percentage of people in IFA group who received one (7.2%) was higher than in the FCA group (2.9%), but this was not statistically significant. These findings are in contrast with a small study from Ruano et al., which suggests that among individuals undergoing 2T termination IOL in the setting of placenta previa, IFA is associated with a smaller difference in pre‐expulsion and post‐expulsion hemoglobin [21]. These findings overall suggest that IFA may be a safe option for patients prior to 2T termination IOL without increasing the risk of maternal health complications.

This study has several important limitations. First, all participating sites were tertiary or quaternary maternal care centers with extensive experience in performing 2T termination IOL between 14 and 27 weeks of gestation. Therefore, the findings may not be generalizable to sites with a lower volume of medication abortions during these gestational age ranges. Second, we were unable to capture the duration between IFA and initiation of IOL; this duration may be important for softening the products of conception and could potentially impact the duration of labor. Given the exclusive use of intracardiac and/or intrathoracic administration of KCl in our dataset, we cannot assess whether there are differential risks for adverse health outcomes based on other substances used for IFA (e.g., lidocaine or digoxin) or route of instillation (i.e., subdiaphragmatic, periumbilical). Additionally, for our secondary outcome of the need for blood transfusion, we did not have information about the specific reason for bleeding (e.g., uterine atony). We also do not have information on the specific indication for use of IFA at each institution, such as gestational duration requirements or patient preference, and this may contribute to selection bias. These factors may play a role in adverse health outcomes and should be evaluated in future research. Finally, due to the small sample size and rare outcome, a small number of patients experienced the secondary composite outcome or the individual components, limiting the ability to see significance. The study was underpowered to assess significant differences between exposure groups.

In summary, 2T termination IOL with IFA was not associated with longer duration of labor nor was it associated with adverse health outcomes when compared to IOL of individuals with FCA. Further research is necessary to address the limitations listed above.

## CONFLICT OF INTEREST STATEMENT

Ashish Premkumar is a scientific consultant for GenBioPro.

## Data Availability

The data that support the findings of this study are available from the corresponding author upon reasonable request.
